# Comprehensive mapping of the cell response to *E. coli* infection in porcine intestinal epithelial cells pretreated with exopolysaccharide derived from *Lactobacillus reuteri*

**DOI:** 10.1186/s13567-020-00773-1

**Published:** 2020-03-31

**Authors:** Ľudmila Tkáčiková, Evelína Mochnáčová, Punit Tyagi, Zuzana Kiššová, Mangesh Bhide

**Affiliations:** 1 Institute of Immunology, University of Veterinary Medicine and Pharmacy in Kosice, Kosice, Slovakia; 2 Laboratory of Biomedical Microbiology and Immunology, University of Veterinary Medicine and Pharmacy in Kosice, Kosice, Slovakia; 3 Institute of Neuroimmunology, SAV, Dubravska cesta 9, Bratislava, Slovakia

## Abstract

Bacterial exopolysaccharides (EPSs) are known to modulate immunity. To date, a plethora of studies have reported the effect of EPSs on intestinal cells; however few works have revealed a complete picture of the signalling events in intestinal epithelial cells induced by bacterial EPSs. Here, using transcriptomics, we comprehensively mapped the biological processes in porcine intestinal epithelial cells challenged with EPS derived from *Lactobacillus reuteri* alone, enterotoxigenic *Escherichia coli* (*ETEC*) or ETEC after pretreatment with EPS. The Gene Ontology analysis of differentially expressed genes (DEGs) showed that ETEC is able to evoke biological processes specifically involved in cell junction reorganization, extracellular matrix degradation, and activation of the innate immune response through the activation of pattern recognition receptors, such as TLRs and CTRs. A total of 495 DEGs were induced in ETEC-challenged cells. On the other hand, EPS pretreatment was able to attenuate overexpression of the genes induced by ETEC infection. The most relevant finding of this study is that EPS has a suppressive effect on the inflammatory response evoked by ETEC infection. On the basis of high-throughput RNA-seq, this report is the first to describe the effects of EPSs derived from *L. reuteri* used as a pretreatment of global gene expression in porcine epithelial cells.

## Introduction

Bacterial exopolysaccharides (EPSs) are extracellular polysaccharides that play pivotal roles in the protection of bacteria and adhesion to host cells. EPSs are either covalently attached as a capsule to the surface of bacteria or released into the environment [1]. Among the beneficial bacteria, *Lactobacillus* represents one of the best producers of EPS. Exopolysaccharides produced by lactobacilli have not only positive effects on their producers [2, 3] but also immunomodulatory effects on the gut mucosal immune system [4–6]. Exopolysaccharides stimulate the immune response in intestinal epithelial cells (IECs) through the activation of C-type lectin receptors (CLRs). The activation of IECs results in the induction of a broad range of cytokines and chemokines, including interleukins, TNF, growth factors and beta-defensins [7]. Thus, IECs play important roles in the activation of dendritic cells that control innate and acquired immune responses [8].

Enterotoxigenic *Escherichia coli* (ETEC) is one of the most common causes of post-weaning diarrhea in pigs [9, 10]. ETEC interacts with epithelial cells, colonizes the small intestine and secretes thermostable (ST) or thermolabile (LT) enterotoxins, inducing acute intestinal diarrhea and inflammation [11]. In addition, ETEC triggers inflammatory responses mediated through other pathogen-associated molecular patterns, such as lipopolysaccharides (LPSs), that significantly contribute to intestinal tissue injury during infection [9, 11]. ETEC infection is responsible for economic losses in the pig industry mainly due to high mortality, morbidity, growth retardation and treatment costs [12]. Thus, it is necessary to protect piglets against ETEC infection by modulating their gut immunity with prebiotics, probiotics or the products of beneficial bacteria such as EPSs.

We have previously observed that the EPS isolated from *Lactobacillus reuteri* strain L26 Biocenol possesses cytokine-modulating activity. This EPS is a homopolymer consisting of α-d-glucose (1→3) and (1→6) glycosidic bonds at a ratio of 1.3:1 with a molecular weight (Mw) of 8.2 × 10^5^ Da [6]. To date, scarce literature is available that presents a complete picture of the cell response (at the transcriptomic level) to the EPS derived from *Lactobacillus.* The plausible protective effect of EPS mediation of the immune response against *E. coli* infection has been documented previously, however, with a small-scale experimental approach [6].

To date, few studies have addressed the cell response to lactobacilli, *E. coli* or bacterial toxins at the transcriptomic level. Kobayashi et al. [13] used a microarray to investigate the response of porcine intestinal epithelial (PIE) cells to the ST toxins of ETEC and *Lactobacillus jensenii*. The transcriptomic response of PIE cells to the viral molecular associated pattern polyinosinic–polycytidylic acid and immunobiotic strains *Lactobacillus rhamnosus* and *Lactobacillus plantarum* was previously studied [14]. Similarly, differences in genome-wide gene expression induced by a mixture of three *Lactobacillus* strains (*L. rhamnosus*, *L. plantarum*, and *Lactobacillus paracasei*) in intestinal porcine epithelial cells (i.e., IPEC-1 cells) were also reported [15]. In another study, gene expression in IPEC-J2 cells exposed to microalgal extracts with or without challenge with ETEC was reported [16]. To our knowledge, no work has been published thus far that maps a complete picture of the IPEC-1 cell response at the transcriptomic level to *E. coli* challenge with or without pretreatment of EPS isolated from the probiotic lactobacilli.

Therefore, the objective of this study was to map a comprehensive picture of the gene expression in IPEC-1 cells challenged with ETEC with or without pretreatment with EPS. We hypothesized that transcriptomic analyses using IPEC-1 cells could provide valuable data to understand the mechanisms involved in the protective effect of EPS mainly against intestinal inflammatory damage caused by *E. coli*.

## Materials and methods

### Bacterial strains

*Lactobacillus reuteri* L26 Biocenol (CCM 8616) was provided by Radomíra Nemcová, Institute of Microbiology and Gnotobiology, University of Veterinary Medicine and Pharmacy in Košice, Slovakia. L26 was cultured in modified de Man-Rogosa-Sharpe medium (MRS; HiMedia, India) containing 10% sucrose (Mikrochem, Slovakia). For EPS extraction, the modified MRS medium was inoculated with overnight culture of *L. reuteri* (10% v/v) and incubated for 48 h at 37 °C.

Hemolytic ETEC strain 11501 (O149: K88^+^, STb^+^, LT^+^, provided by Martin Faldyna, Veterinary Research Institute, Brno, Czech Republic) was cultivated overnight in lysogeny broth (LB; Sigma-Aldrich, USA) at 37 °C with constant shaking (100 rpm). The LB was inoculated with 1% (v/v) of a 16-h ETEC culture and incubated for the next 3 h at 37 °C with constant shaking. The number of *E. coli* was determined using a spectrophotometer at OD_600_ based on the following equation: OD_600_ of 1.0 = 8 × 10^8^ cells/mL.

### Extraction and purification of the EPSs

The EPS was isolated and purified from *L. reuteri* L26 exactly as described in our previous report [6].

### IPEC-1 cell culture and experimental design

The IPEC-1 cell line was grown in 6-well culture plates in medium containing Dulbecco’s modified Eagle medium/F-12 (Sigma-Aldrich) supplemented with 5% foetal bovine serum (FBS; Lonza, Switzerland), 5 ng/mL epidermal growth factor (BD Biosciences, USA), 10 μg/mL insulin, 10 μg/mL transferrin, and 10 ng/mL selenium (Lonza) at 37 °C in a fully humidified atmosphere with 5% CO_2_. Upon reaching 70% confluency, the cells were washed with sterile phosphate-buffered saline, and EPS (0.1 mg/mL, 1 mL each well) reconstituted in IPEC-1 cell medium without FBS was added. After 4 h of preincubation with the EPS, the cells were challenged with ETEC (multiplicity of infection: 50:1) without refreshing of the medium for 45 min. Subsequently, the monolayers were washed with sterile PBS and stored at −20 °C until further use.

### RNA isolation and integrity

mRNA from the IPEC-1 cells was isolated using an RNeasy Mini kit (Qiagen, Germany) according to the manufacturer’s instructions. DNaseI (Qiagen) treatment was incorporated during RNA isolation. The integrity of the RNA was monitored using capillary electrophoresis (Fragment Analyzer, Advanced Analytical Technologies, Inc., USA).

### Preparation of the library

A total of 250 ng of RNA was reverse transcribed with oligo-dT primers for the synthesis of first-strand cDNA using a QuantSeq 3′ mRNA-seq library prep kit (Lexogen, Austria). All the steps described below were completed exactly following the manufacturer’s instructions. The RNA template was removed with RNA removal solution (RS buffer, Lexogen), and the second strand was synthesized using a random hexamer primer that contains Illumina-compatible linker sequences at its 5′ end. The double-stranded DNA libraries were purified using magnetic beads provided in the kit. Each library was amplified by PCR using unique, single-indexing i7 primers to add the complete adapter sequence required for cluster generation and to generate sufficient DNA for sequencing and quality control. The number of PCR cycles for each library was determined using a PCR Add-on Kit for Illumina (Lexogen). The cycles used for library amplification were as follows: IPEC-1 cells challenged with ETEC—20 cycles, IPEC-1 cells treated with EPS—20 cycles, IPEC-1 cells pretreated with EPS and challenged with ETEC—20 cycles, and non-treated cells—17 cycles. Amplified libraries were purified using the magnetic beads supplied in the kit. The quality of the libraries and length of the fragments were determined on a fragment analyser.

### NGS sequencing

Libraries were sequenced on a Illumina NextSeq, single-end, 75 bp, to a minimal depth of 8 million reads per sample. FASTQ files were processed and aligned to the reference genome using STAR alignment software [17]. The preprocessing includes adaptor trimming and removal of the initial 10 bases (recommended for QuantSeq as these bases are random priming sites). Reads were counted with STAR V 2.5.2b. To perform differential gene expression analysis, edgeR, the open source R package, version 3.12 was used [18]. Low read counts, with less than 3 CPM (count per million), were filtered out using the filterByExp function of the edgeR package. The identification of differentially expressed genes (DEGs) was accomplished by using the glmTreat and glmQLFit (quasi-likelihood, QL) functions of edgeR in the R package, considering log fold change (logFC) values greater than ± 1.2 and FDR values less than 0.05.

### Quantitative expression analysis by RT-PCR

RNA was reverse transcribed into cDNA using random hexamers (Thermo Fisher Scientific). Briefly, 1 μg of RNA and 100 pMol of random hexamers were mixed and incubated for 5 min at 65 °C. Subsequently, 4 μL of 5× reaction buffer, 2 μL of dNTP (10 mM), 1 μL of RevertAid reverse transcriptase (200 U) (Thermo Fisher Scientific, USA) and 0.5 μL RiboLock RNase inhibitor (20 U) (Thermo Fisher Scientific) were added. The reaction mixture was incubated for 10 min at 25 °C, 1 h at 42 °C, and 10 min at 70 °C.

Primers used in qRT-PCR were designed using Geneious Pro software (Biomatters, USA); they are presented in Table 1. The reaction mix of qRT-PCR consisted of 6 ng of cDNA, 1 × iQ SYBR Green Supermix (Bio-Rad, Hercules, CA), gene-specific primers (12.5 pMol each) and RNase-free water to a total volume of 20 μL. Each reaction was performed in triplicate. The amplification cycles were as follows: 95 °C − 10 min, 35 × [95 °C – 30 s., 55–60 °C – 30 s (annealing temperature varied according to the primers used), 72  °C for 30 s (signal capture)], melting curve from 60 to 95  °C – 0.3% temperature increment/s. (StepOnePlus, Thermo Fisher Scientific, USA). The gene expression (ΔΔCt) was normalized to that of the housekeeping gene, β-2-microglobulin (B2M). The ΔΔCt values were converted to logFC values using an online server [19]. The expression values for the DEGs obtained from RNA-seq and qRT-PCR were correlated with Pearson correlation coefficients (PCCs) using an online server [20].

**Table 1:** **PCR primers used in this study**

Genes	Forward primer (5′→3′)	Reverse primer (5′→3′)	T_m_ (°C)	Product length (bp)	Primer designed using sequence from NCBI repository GeneID
B2M	CCGCATCTCCGTGTACTACAA	CAGCCCCTTCTGTATAGTGGC	55/60	197	100153507
AHCYL	TTGACCCCATCTGTGCTCTG	TGGCCCATATTGCACACGAT	55	172	100512899
NFKB1	TCGCTGCCAAAGAAGGACAT	TAGCGTTCAGACCTTCACCG	55	102	751869
TRPC1	GCCTCCGACATTCCAGGTTT	TACATTGCCGGGCTAGTTCC	55	180	100156938
YWHAZ	CCCAGAGAAAGCCTGCTCTC	TTCCCCTCCTTCTCCTGCTT	55	181	780440
PSME1	AGTATTTCTCTGAGCGGGGC	ATCCCGGTACTCTGCCTCAT	55	107	397572
EHMT2	AAGTGCAGCATTTCCGCATG	GAACCCAACTCCTCCGACAG	60	115	100124382

### Data analysis

The logical relation of DEGs in the group with a negative control background was calculated using Excel (MS office), and a Venn diagram was constructed. To group DEGs into GO biological processes, the Reactome server was used [21], and to construct heat maps, the Heatmapper server was used [22]. Signalling pathways were downloaded from the KEGG server [23], and the DEGs involved in the KEGG pathways were manually highlighted.

## Results

### Differentially expressed genes and validation

**Figure 1:**
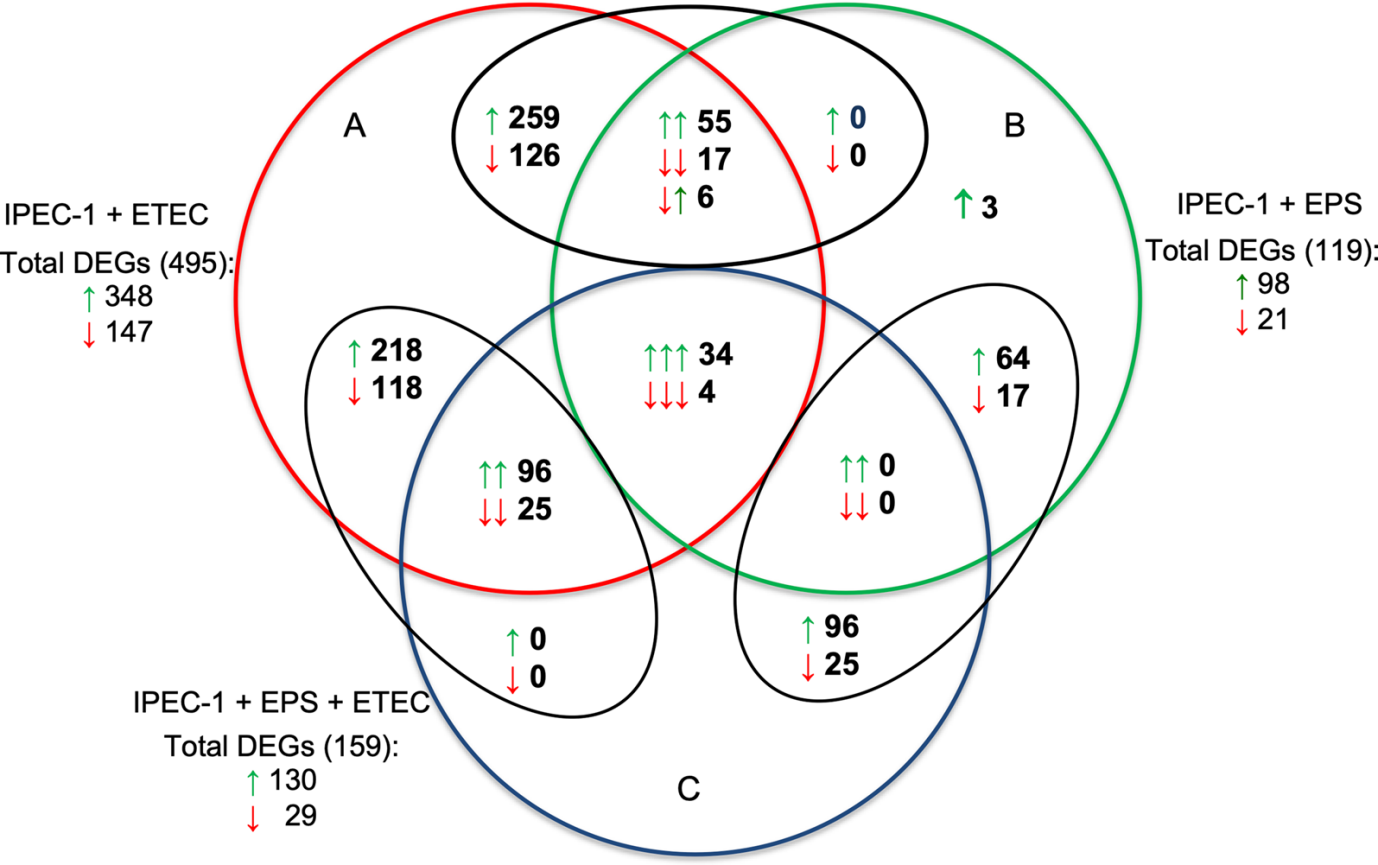
**Venn diagram presenting the number of DEGs in the IPEC-1 cells challenged with ETEC (A), treated with EPS (B), or pretreated with EPS before ETEC challenge (C).** Green arrows—upregulated DEGs. Red arrows—downregulated DEGs.

In total, 495 genes were differentially expressed in the IPEC-1 cells challenged with ETEC, among which 348 (70.3%) genes were upregulated and 147 (29.7%) were downregulated (Figure 1, Additional file 8: Data set 1.1). A total of 119 genes were found to be differentially expressed in EPS-treated cells (98 genes upregulated and 21 genes downregulated) (Figure 1, Additional file 8: Data set 1.2). In the IPEC-1 cells treated with EPS and then challenged with ETEC, 130 (81.8%) genes were upregulated, and 29 (18.2%) genes were downregulated (Table 2, Figure 1 and Additional file 8: Data set 1.3). It is important to note that 336 genes were differentially expressed in the *E. coli*-infected cells that were not found to be differentially expressed in the cells pretreated and then challenged with *E. coli*. It is also noteworthy that 3 genes (Wnt inhibitory factor 1, Zinc finger CCCH-type antiviral protein 1 isoform 1 and Zinc finger protein 181 isoform X2) were uniquely expressed among the cells treated only with EPS (Figure 1, Additional file 8: Data set 1.2). The common DEGs found among the cells challenged with different treatments are listed in Additional file 8: Data sets 1.4 to 1.6. When searching for common DEGS in all the cells subjected to one of the three treatments, we found only 38 DEGs (34 upregulated and 4 downregulated genes) (Figure 1, Additional file 8: Data set 1.7).

**Table 2:** **Number of DEGs found in the cells induced with different treatments**

	Total found DEGs	Upregulated		Downregulated	
		Number of DEGs	%	Number of DEGs	%
IPEC-1 cells challenged with ETEC	495	348	70.3	147	29.7
IPEC-1 cells treated with EPS	119	98	82.4	21	17.6
IPEC-1 cells pretreated with EPS and then challenged with ETEC	159	130	81.8	29	18.2

**Figure 2:**
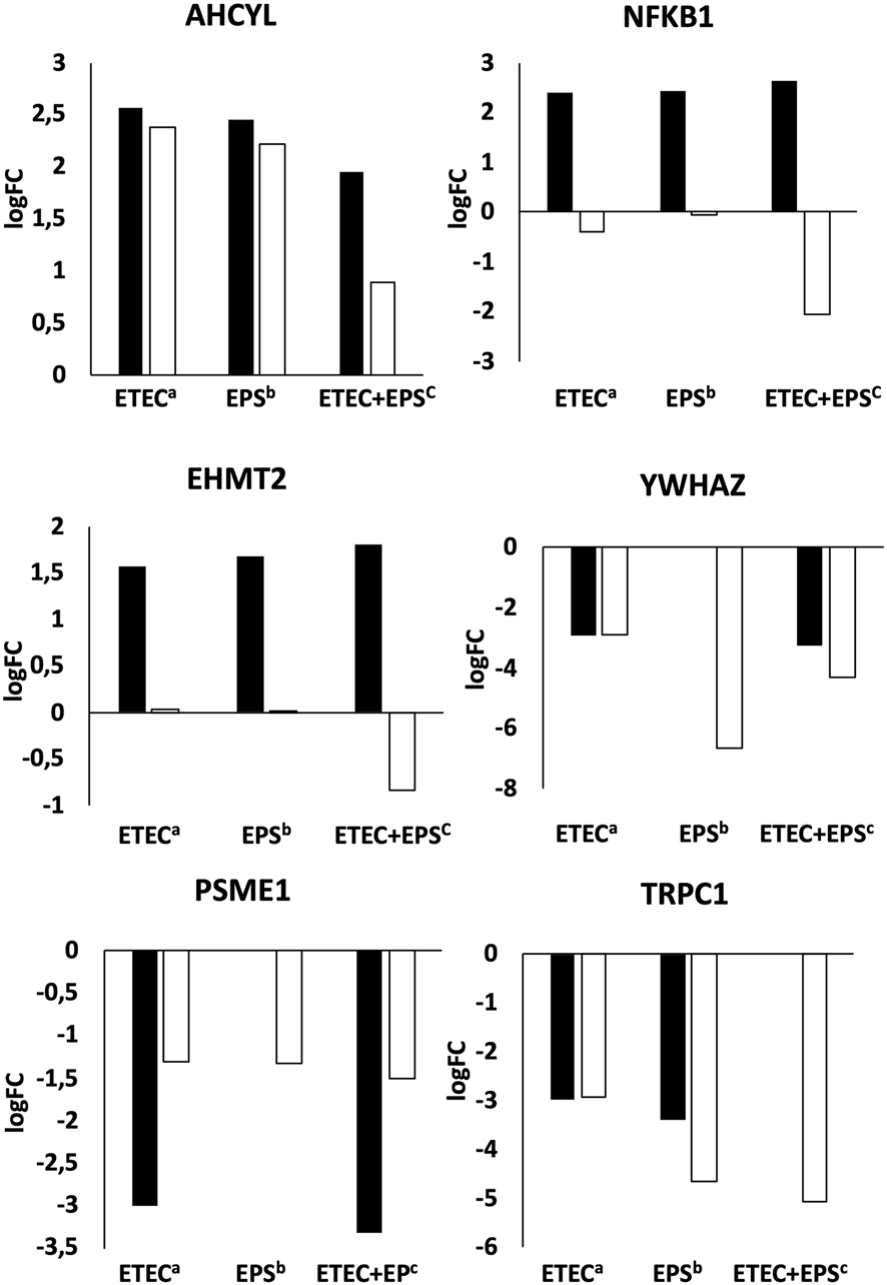
**Validation of DEGs with qRT-PCR. IPEC-1 cells were challenged with ETEC, treated with EPS, or pretreated with EPS before ETEC challenge.** Black bars—logFC values from RNA-seq; white bars—logFC values calculated from qRT-PCR. Pearson correlation coefficient (PCC) (r = 0.712 for the IPEC-1 cells exposed to ETEC^a^; r = 0.728 for the IPEC-1 cells exposed to EPS^b^; and r = 0.455 for the IPEC-1 cells exposed to EPS and ETEC^c^; *p* < 0.01). Note that standard deviation (SD) is not shown here, as the expression levels (log^2^ of the ΔΔCT values) of the DEGs in qRT-PCR was calculated based on average CT values of triplicates (the SD of CT ranged between 0.005 and 0.28).

To validate the results obtained from RNA-seq, the differential expression of 6 representative genes was analysed with qRT-PCR. The results obtained from both techniques were consistent (Figure 2) as determined ompared with Pearson correlation coefficient (PCC) (r = 0.712 for IPEC-1 cells exposed to ETEC; r = 0.728 for IPEC-1 cells exposed to EPS; and r = 0.455 for IPEC-1 cells exposed to EPS and ETEC; *p* < 0.01). Following validation of the results, DEGs were segregated according to the GO biological process category using a peer-reviewed server—Reactome [21].

The results from segregating the DEGs according to GO biological process category, performed with the Reactome server, are presented in Additional file 1. Treatment with *E. coli* or EPS caused the induction of genes involved in several pathways (pathway identifiers are listed in Additional file 8: Data sets 1.8 to 1.10); however, here, we present the pathways that were directly related to the pathogenies of ETEC. These pathways are involved in cell junction organization, extracellular matrix (ECM) organization, the innate immune response, the CLR-related pathway, TLR cascades and cytokine signalling.

### DEGs involved in cell junction organization

**Figure 3:**
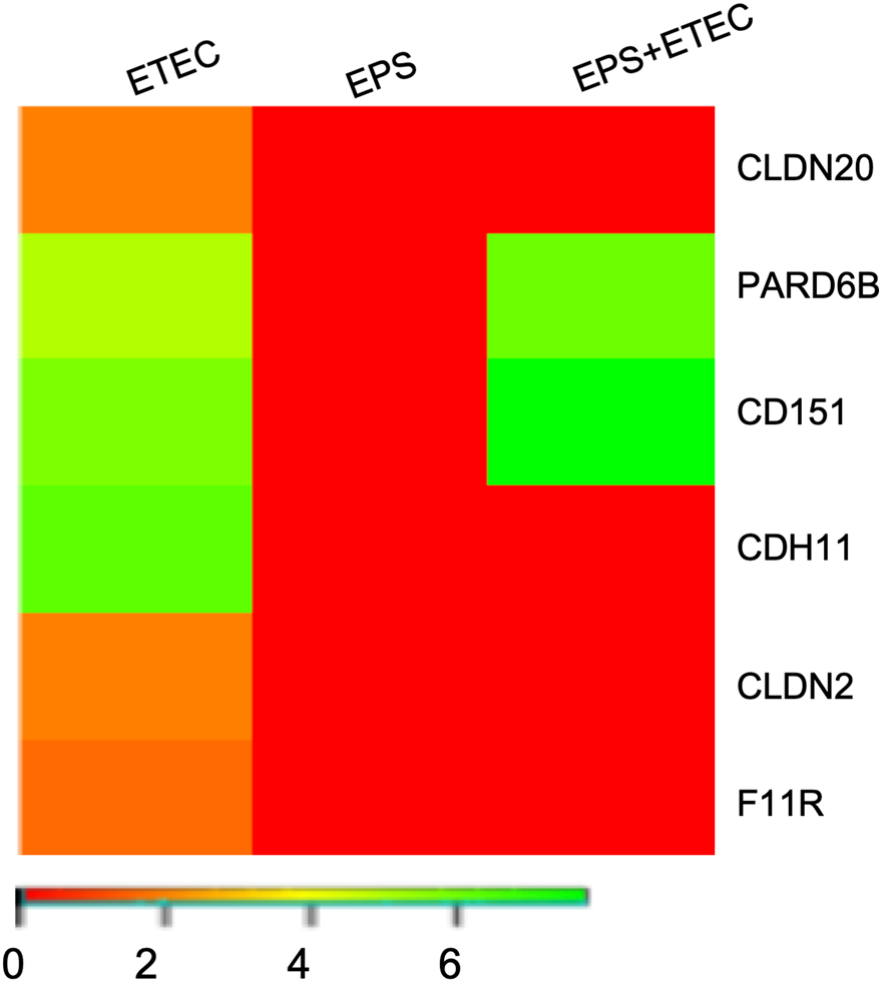
**DEGs related to the GO biological process “Cell junction organization”.** Heat map showing the DEGs in the IPEC-1 cells challenged with ETEC, treated with EPS, or pretreated with EPS before ETEC challenge. The bar shows logFC values with the corresponding colour code.

The intestinal epithelial barrier plays an essential role in host defence against infections. The epithelial cell–cell junctional system comprises adherent junctions (AJs), tight junctions (TJs) and desmosomes [24], the disruption of which leads to an increase in the permeability of the barrier. Enterotoxins produced by ETEC strains may cause morphological changes in the intestinal mucosa, including the reorganization of cellular junctional proteins; thus, DEGs involved in this biological process are of main importance for this study. Six genes (CLDN20, PARD6B, CD151, CDH11, CLDN2 and F11R) related to the GO biological process “Cell junction organization” were upregulated by *E. coli* infection (Figure 3), whereas these genes were not induced by EPS (Additional file 2). A significant upregulation of CDH11 (logFC of 6.33), encoding cell adhesion protein cadherin 11, observed in the cells infected with *E. coli* was abolished by EPS pretreatment before ETEC challenge. Cadherin 11 participates in the formation of AJs as is CLDN2 and CLDN20 (both logFC of 1.95). EPS pretreatment also caused the upregulation of F11R (encoding junctional adhesion molecule A, JAM-A) only in the infected cells without EPS pretreatment (logFC of 1.66). JAM-A is an important molecule in the formation of epithelial TJs [25].

Notably, EPS pretreatment before ETEC challenge induced the expression of CD151 (logFC of 7.76) and PARD6B (logFC of 6.14) genes. CD151 (a tetraspanin) participates in the formation of hemidesmosomes, the specialized multiprotein junctional complex that connects the keratin cytoskeleton of epithelial cells to the extracellular matrix and plays a critical role in the maintenance of tissue structure [26], while PARD6B (Par-6 family cell polarity regulator beta) participates in the formation of TJs [27].

### DEGs involved in extracellular matrix (ECM) organization

**Figure 4:**
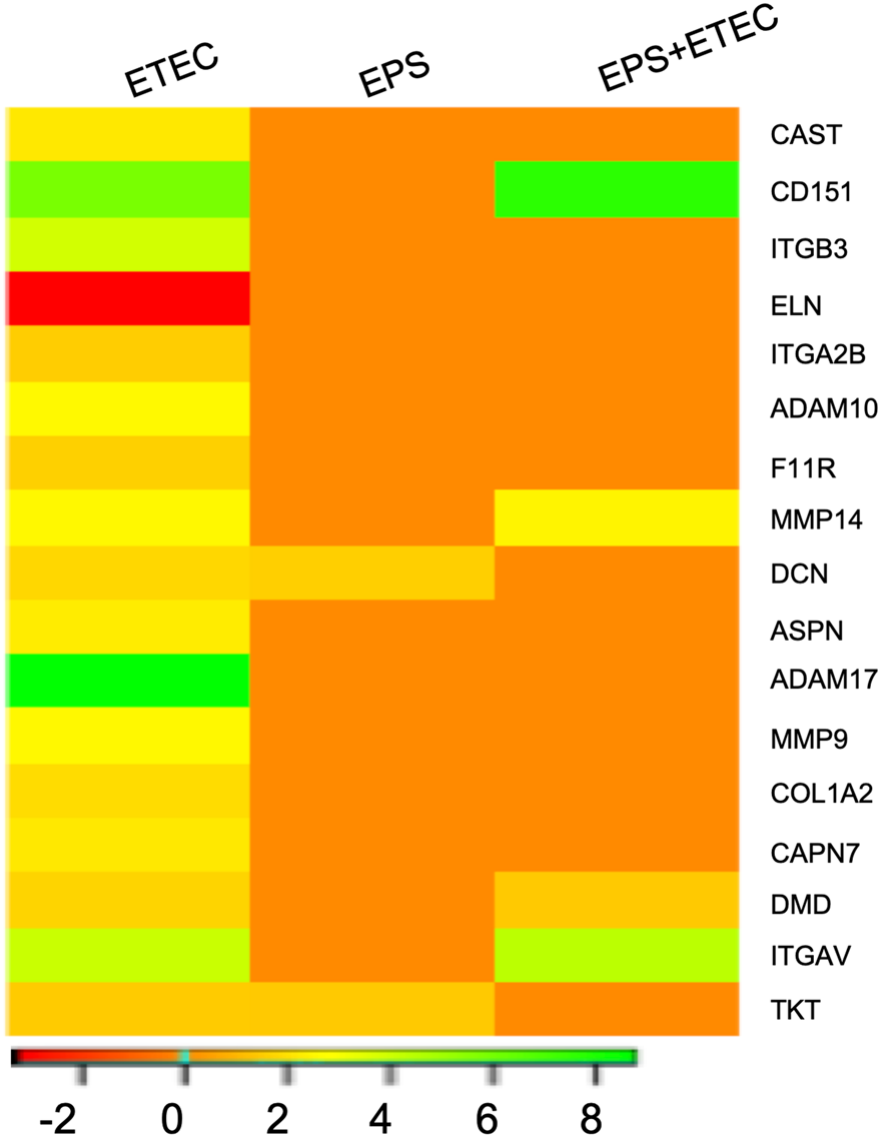
**DEGs related to the GO biological process “Extracellular matrix organization”.** Heat map showing the DEGs in the IPEC-1 cells challenged with ETEC, treated with EPS, or pretreated with EPS before ETEC challenge. The bar shows the logFC values with the corresponding colour code.

ETEC enterotoxins cause morphological changes in the intestinal mucosa; thus, the regulation of the genes related to the GO biological process “Extracellular matrix organization” were analysed (Figure 4). Seventeen genes were induced in this GO process. *E. coli* infection resulted in the upregulation of the genes encoding proteases, which participate in the degradation of ECM, namely, ADAM10 (logFC of 2.62), ADAM17 (logFC of 8.75), MMP9 and MMP14 (both with a logFC of 2.58), CAPN7 and CAST (both with logFC of 2.20). The expression of these proteases remained unchanged after EPS pretreatment prior to *E. coli* infection, except MMP14 (logFC of 2.47). This finding indicates that the EPS pretreatment reduced the tissue damage caused by the ETEC infection. Although the genes involved in the degradation of ECM were upregulated, the expression of the ELN and COL1A2 genes encoding structural components of the ECM was changed only in the cells challenged with ETEC (Figure 4, Additional file 3). The ELN gene, encoding elastin, which contributes to the structural integrity of the ECM, was downregulated (logFC of −3.29), and the collagen type I alpha 2 chain-encoding gene, COL1A2, was upregulated (logFC of 1.90). Two small leucine-rich proteoglycans (SLRPs), decorin and asporin, encoded by the DCN and ASPN genes, respectively, were induced in the EPS-treated cells. SLRPs interact with different cell surface receptors, cytokines, growth factors and other ECM components, leading to the modulation of cellular functions. Decorin interacts with collagen and acts as a sink for all three isoforms of TGF-beta [28]. Degradation of decorin by matrix metalloproteinases results in the release of TGF-beta [29]. DCN was upregulated in the *E. coli*-infected cells (logFC of 1.81) and EPS-treated cells (logFC of 1.59), whereas ASPN was upregulated only in the cells challenged with ETEC (logFC of 2.30) (Figure 4). The expression of three integrins (ITGAV, ITGB3 and ITGA2B) was altered in the *E. coli*-infected cells (Figure 4). Integrins are heterodimeric transmembrane receptors that mediate cell adhesion and bind extracellular matrix glycoproteins such as laminins and collagens in basement membranes or connective tissue components such as fibronectin [30]. Two of these genes, ITGB3 (logFC of 3.77) and ITGA2B (logFC of 1.58), were upregulated in the cells challenged with ETEC, while this upregulation was abolished by pretreating cells with EPS before infection (Figure 4). Such attenuation was not observed in the case of the ITGAV gene in the pretreated cells (ETEC—logFC of 3.99 after pretreatment and ETEC—logFC of 4.35).

### DEGs related to innate immune response

**Figure 5:**
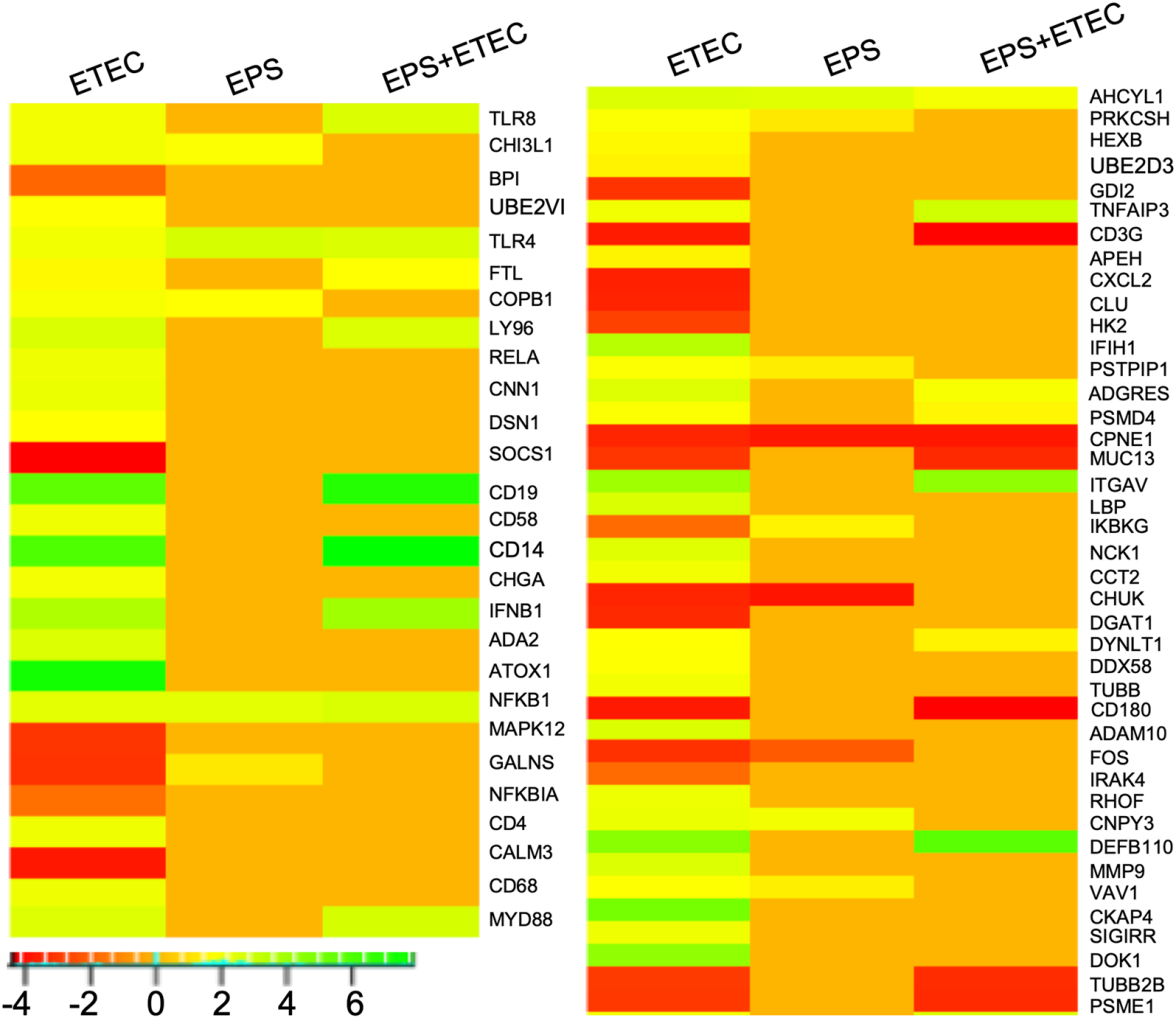
**DEGs related to the GO biological process “Innate immune system”.** Heat map showing the DEGs in the IPEC-1 cells challenged with ETEC, treated with EPS, or pretreated with EPS before ETEC challenge. The bar shows logFC values with the corresponding colour code.

In total, 68 genes (21 downregulated and 47 upregulated) categorized in the GO biological process “Innate immune system” were induced by ETEC challenge (Figure 5). On the other hand, ETEC challenge induced only 22 genes (6 downregulated and 16 upregulated) in the EPS-pretreated cells. In the cells treated only with EPS, 14 genes (3 downregulated and 11 upregulated) were found with altered expression. In the *E. coli*-infected cells, the highest fold-change was observed for ATOX1 (logFC of 7.55), encoding antioxidant 1 copper chaperone, which binds and delivers cytosolic copper to the copper-ATPase proteins [31]. The SOCS1 gene, encoding suppressor of cytokine signaling 1, was the most downregulated (logFC of -4.38); SOCS1 is involved in the negative regulation of cytokines that signal through the JAK/STAT3 pathway. Expression of the ATOX1 and SOCS1 genes was abolished by the EPS treatment and ETEC challenge of in the pretreated cells (Figure 5).

In the cells pretreated with EPS and then infected with *E. coli*, the highest fold change was observed for the gene encoding CD14 (logFC of 7.87), while the gene encoding CD180 was the downregulated to the greatest extent (logFC of −4.26). The CD14 molecule is a coreceptor for bacterial LPS [32], and CD180 (also known as RP105) is a negative regulator of TLR4 [5]. The expression of these genes was also altered in the *E. coli*-infected cells (CD14, logFC of 5.95; CD180, logFC of −3.73) but not in the cells treated only with EPS. Notably, treatment of the cells only with EPS induced TLR4 (logFC of 2.77). The expression of this gene was also upregulated in ETEC-challenged cells (both without pretreatment, logFC of 2.06, or with pretreatment, logFC of 2.62). The gene encoding CHUK, a component in the inhibitor of the nuclear factor kappa B kinase complex, was the downregulated to the greatest extent (logFC of −3.85). The expression of the CHUK gene was also downregulated in the ETEC-challenged cells (logFC of −3.47) but not in the cells pretreated with EPS and infected with ETEC (Figure 5).

### DEGs related to C-type lectin receptors

**Figure 6:**
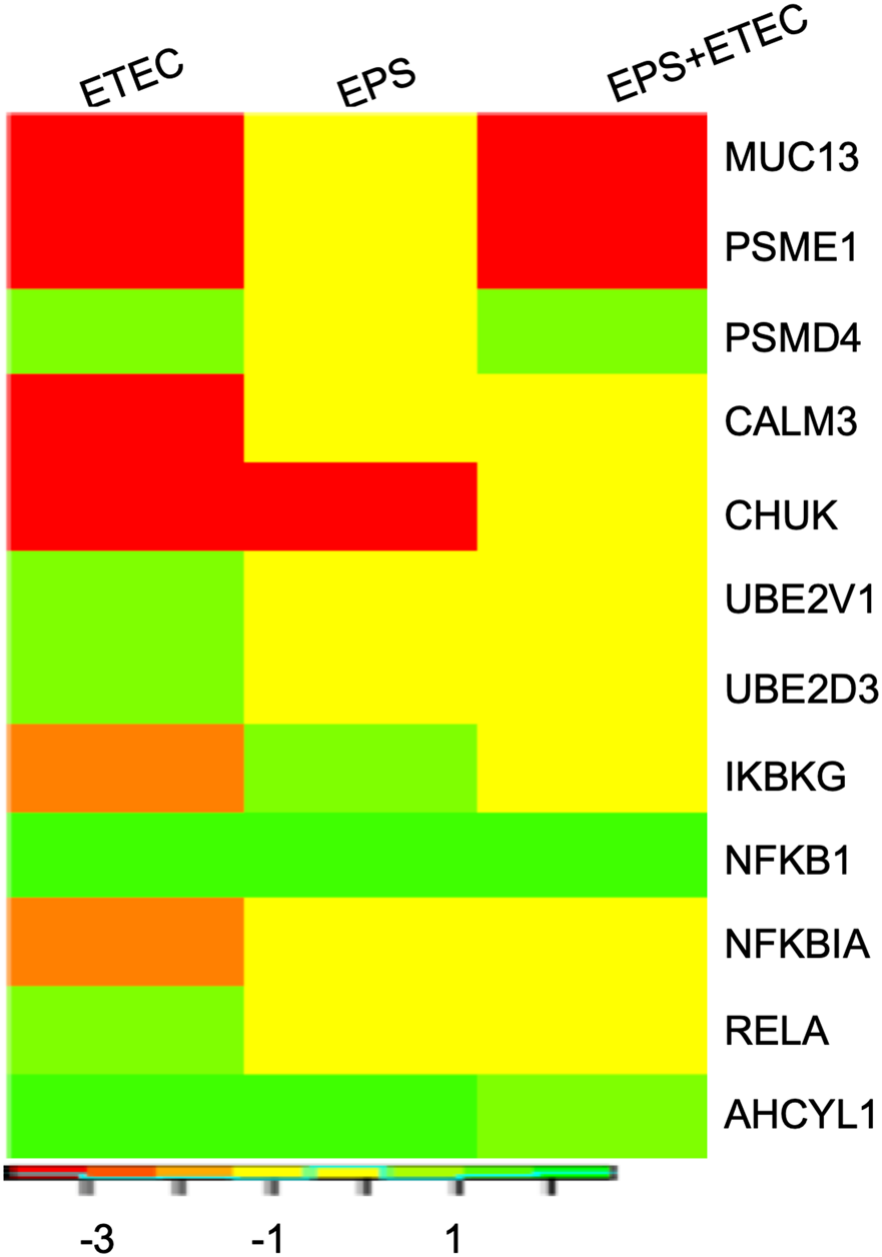
**DEGs related to the GO biological process “CLR”.** Heat map showing the DEGs in the IPEC-1 cells challenged with ETEC, treated with EPS, or pretreated with EPS before ETEC challenge. The bar shows logFC values with the corresponding colour code.

C-type lectin receptors (CLRs) constitute a large superfamily of proteins that act as pattern-recognition receptors for pathogen-derived carbohydrates. Twelve DEGs (6 downregulated and 6 upregulated) related to the GO biological process “C-type lectin receptors” were induced by ETEC infection (Figure 6). Two genes, AHCYL1 (adenosylhomocysteinase-like 1) and CALM3 (calmodulin 3), which are involved in the dectin-1-dependent calcineurin/NFAT pathway, were altered (AHCYL1, logFC of 2.57, and CALM3, logFC of −3.78). Activation of the AHCYL1 gene in all experimental groups was observed (Figure 6). AHCYL1 interacts with the inositol IP3 receptor and is involved in intracellular calcium release [33, 34]. Interestingly, the CALM3 gene was downregulated only in cells challenged with *E. coli*; hence, calmodulin could not activate calcineurin, and therefore, the transcription factor NFAT was not activated. This downregulation of the CALM3 gene was abolished by EPS pretreatment administered prior to ETEC infection.

Seven DEGs (NFKBIA, RELA, UBE2D3, UBE2V1, IKBKG, CHUK, and NFKB1) that participate in Dectin-1-mediated activation of NF-κB were found to be induced (Figure 6, Additional file 4). EPS treatment induced alterations in IKBKG, CHUK and NFKB1, while EPS pretreatment induced the upregulation of NFKB1 (Figure 6). The ETEC challenge induces the expression of the UBE2D3 and UBE2V1 genes (both with logFC of 1.48) encoding ubiquitin-conjugating enzyme E2. The UBE2D3 enzyme participates in the polyubiquitination of NF-kappa-B inhibitor alpha (IKBA), while the UBE2V1 enzyme forms a heterodimer complex with UBE2N (UBE2V1-UBE2N), which together with TRAF6, participates in the polyubiquitination of NF-kappa-B inhibitor gamma (IKBKG). Ubiquitination of inhibitors leads to the subsequent proteasomal degradation of the inhibitors; therefore, the upregulation of the UBE2D3 and UBE2V1 genes likely promotes the release of the NF-κB transcription factor [35]. The expression of these genes was unaltered in the EPS-treated cells and the pretreated cells challenged with ETEC. We also found downregulation of the NFKBIA gene encoding IKBA in the cells infected with ETEC (logFC of −1.71). This downregulation was abolished by EPS pretreatment prior to *E. coli* infection. The downregulation of NFKBIA was associated with the upregulation of the genes encoding two subunits of NF-κB: NFKB1, encoding the p105 subunit (logFC of 2.40), and RELA, encoding the p65 subunit (logFC of 2.13), in cells infected with *E. coli* (Additional file 5). The expression of the p105 subunit was also increased in cells treated with EPS (logFC of 2.42) or pretreated before the *E. coli* challenge (logFC of 2.65); however, in this case, the expression of the p65 subunit was unaffected.

### DEGs related to TLR cascades

**Figure 7:**
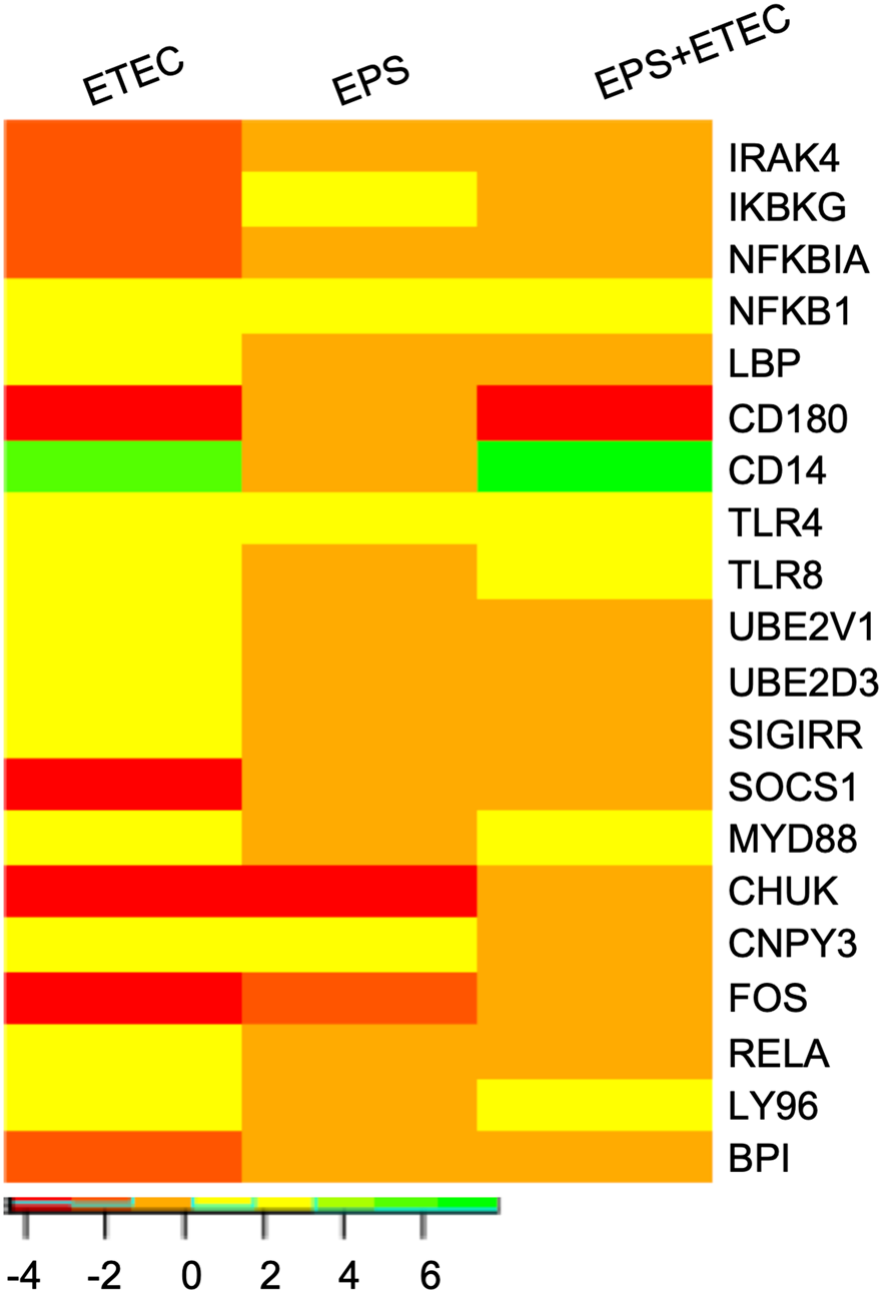
**DEGs related to the GO biological process “TLR cascades”.** Heat map showing the DEGs in the IPEC-1 cells challenged with ETEC, treated with EPS, or pretreated with EPS before ETEC challenge. The bar shows logFC values with the corresponding colour code.

In the cells infected with *E. coli,* 20 induced DEGs (8 downregulated and 12 upregulated) t are related to the GO biological process “Toll-like receptor cascades” (Figure 7). TLRs are major microbial pattern-recognition receptors. Eighteen DEGs (8 downregulated and 10 upregulated) were associated with the GO biological process “TLR4 cascade”, and 14 DEGs (6 downregulated and 8 upregulated) were categorized in the “TLR2 cascade” process (Additional file 6). Although TLR4 was upregulated in each experimental group, the LBP gene encoding LPS-binding protein (LBP) was induced only in the ETEC-challenged cells (logFC of 2.65). On the other hand, the expression of CD14- and Ly96 (also known as MD-2)-encoding genes was upregulated only after the ETEC challenge, irrespective of pretreatment with EPS.

The binding of a ligand to TLR4 initiates MyD88-dependent and TRIF-dependent pathways [36]. Expression of the MyD88 gene was upregulated only in the case of ETEC challenge (with and without pretreatment, logFC of 2.54 and logFC of 2.75, respectively). The activation of the MyD88-dependent pathway causes the activation of TAK1 (transforming growth factor (TGF)-activating kinase 1); however, we did not observe altered expression of the genes participating in the activation of TAK1, except for the downregulation of IRAK-4 (which encodes IL-1R-associated kinase 4) in the ETEC-challenged cells (logFC of −1.77). Activated TAK1 phosphorylates the IκB kinase (IKK) complex critical for the phosphorylation of the inhibitory protein IκB subunit of the NF-κB transcription factor. Three genes associated with the regulation of NF-κB were downregulated in the cells challenged with *E. coli*, namely, NFKBIA, encoding the IκBα inhibitor of NF-κB (logFC of −1.71); CHUK, encoding the IKKα kinase subunit (logFC of −3.47); and IKBKG, encoding the IKKγ kinase subunit (logFC of −1.79). Notably, the expression of these genes was unaltered in the cells pretreated with EPS and then challenged with *E. coli* (Figure 7). The ETEC challenge also induced the expression of the UBE2D3 and UBE2V1 genes (both with logFC of 1.48), which are involved in the GO biological process “MyD88-independent pathway”. The expression of these genes was unaltered in the EPS-treated and pretreated cells challenged with ETEC.

Various negative regulatory mechanisms are necessary to attenuate TLR4 signalling and maintain immune system balance, including the activity of the TLR4-negative regulators CD180 (also known as RP105), SIGIRR (single Ig IL‐1 receptor‐related molecule), TNFAIP3 (TNF alpha-induced protein 3, also called A20) and SOCS1 (suppressor of cytokine signaling 1). The expression of CD180 was downregulated only in the case of ETEC challenge (with and without pretreatment, logFC of −4.26 and logFC of −3.73, respectively), whereas treatment with only EPS had no effect on their expression levels. On the other hand, the expression of SIGIRR was induced in *E. coli*-infected cells (logFC of 2.09). The expression of TNFAIP3 was upregulated in the *E. coli*-challenged cells (with and without pretreatment, logFC of 2.90 and logFC of 2.09, respectively). The expression of SOCS1 was altered only in the ETEC-challenged cells (logFC of −4.38) (Figure 7).

### DEGs related to cytokine signalling

**Figure 8:**
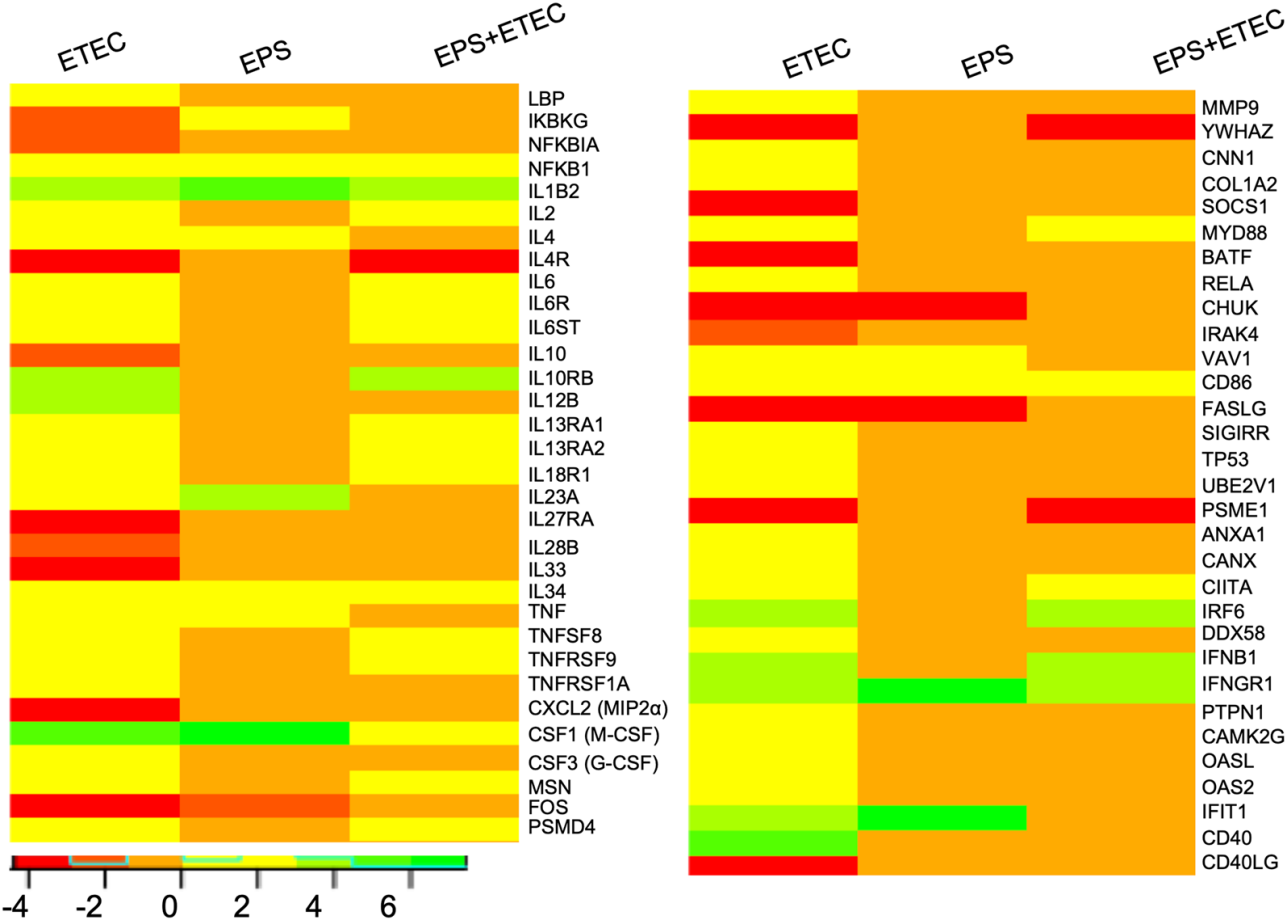
**DEGs related to the GO biological process “Cytokine signaling in immune system”.** Heat map showing the DEGs in the IPEC-1 cells challenged with ETEC, treated with EPS, or pretreated with EPS before ETEC challenge. The bar shows logFC values with the corresponding colour code.

Studying the ETEC-challenged cells, we observed 73 DEGs (17 downregulated and 56 upregulated) related to the GO biological process “Cytokine signaling in immune system” (Figure 8). As expected, *E. coli* infection of IPEC-1 cells induced the upregulation of genes encoding cytokines participating in the inflammatory response, such as IL1B2 (logFC of 3.07), TNF (logFC of 2.01), IL6 (logFC of 2.73), IL2 (logFC of 2.86), IL4 (logFC of 1.46), IL12b (logFC of 2.86) and IL23A (logFC of 2.79) (Additional file 7). It is noteworthy that the expression of the TNF, IL12b, and IL4 genes was unaltered in the cells pretreated with EPS (Figure 8), while other the ILs were upregulated: IL1B2 (logFC of 4.94), TNF (logFC of 2.59), IL4 (logFC of 1.59) and IL23A (logFC of 3.03). In all the experimental groups, we observed the upregulation of macrophage colony-stimulating factor 1 (CSF1) and IL34, which play essential roles in the proliferation and differentiation of monocytes and macrophages. The upregulation of CSF1 in the EPS-treated cells found have an logFC value of 7.44. It is important to note that administration of the EPS treatment prior to *E. coli* infection attenuated CSF1 gene expression, logFC of 1.70. On the other hand, no attenuating effect of the EPS pretreatment on IFNB1 (encoding interferon beta 1) was found. The logFC of this gene was 3.63 in the cells challenged with *E. coli*, while the logFC was 4.00 in the cells pretreated before infection.

The ETEC infection of IPEC-1 cells induced the downregulation of the CXCL2 gene (logFC of −3.59), encoding a powerful neutrophil chemoattractant, and the IL10 gene (logFC of −1.77), encoding interleukin 10, which has powerful anti-inflammatory functions (Figure 8). The expression of these genes was unaltered in the EPS-pretreated cells challenged with ETEC, suggesting that EPS may enhance the pro-inflammatory response during *E. coli* infection. The ETEC challenge of EPS-treated and untreated cells also induced upregulation of genes encoding receptors for some cytokines, such as IL6R, IL10RB, IL13RA, IL18R1, TNFRSF1A and TNFRSF9. The expression of these genes was unaltered in the EPS-treated and pretreated cells challenged with ETEC (Figure 8).

The *E. coli* infection of IPEC-1 cells induced the upregulation of the IFNB1 gene encoding interferon beta 1 (logFC of 3.63) and the IFNGR1 gene (logFC of 3.55), encoding the ligand-binding alpha chain of the gamma interferon receptor. Expression of the IFNB1 gene was upregulated in the EPS-pretreated cells challenged with ETEC (logFC 4.00) but was unaltered in the EPS-treated cells. The IFNGR1 gene was upregulated in all experimental groups, with expression level highest in the EPS-treated cells (logFC of 6.84) (Figure 8).

## Discussion

﻿ETECs adhere to epithelial cells through the interaction of fimbrial and non-fimbrial adhesins with cell surface receptors. ﻿Upon attachment, they release LT and/or ST enterotoxins, which act upon intestinal enterocytes by disrupting electrolyte homeostasis, resulting in fluid loss and eventually secretory diarrhea [37, 38]. LTs activate adenylate cyclase, which substantially increases the intracellular concentration of cAMP [39]. The increased concentration of cAMP leads to the activation of cAMP-dependent kinase protein, which then stimulates chloride channels and inhibits Na^+^ uptake [9]. In the current study, in *E. coli*-infected cells, the expression of adenylate cyclase-activating polypeptide (ADCYAP1 gene) was downregulated, while the ADCYAP1R1 gene encoding the receptor for ADCYAP1 was upregulated in all experimental groups (Additional file 8: Data set 1.7). Notably, some of the cAMP-dependent kinase proteins (PRKAG3 and PRKAR1A) were upregulated in the *E. coli*-infected cells, while EPS pretreatment before infection abolished the induction of PRKAR1A (Additional file 8: Data set 1.5). STb, one of the two classes of heat stable toxins [9, 30], stimulates a GTP-binding regulatory protein, resulting in an increase in intracellular Ca^2+^ levels activating Ca^2+^/calmodulin-dependent protein kinase II (CAMKII), which opens a calcium-activated chloride channel. *E. coli* induced the regulation of CAMKII (CAMK2G); however, the upregulation was attenuated by EPS pretreatment (Figure 8). Thus, EPS pretreatment can reduce the overexpression of PRKAR1A and CAMK2G, which are activated in *E. coli* infection. Considering the crucial role of PRKAR1A and CAMK2G in electrolyte loss (mediated by the activation of ion channels), it is tempting to speculate that suppression of the induction of both genes by the EPS of *L. reuteri* prevents the development of diarrhea in ETEC-infected piglets. A similar effect was reported in a recent report, wherein authors demonstrated that pretreatment of IPEC-J2 cells with various strains of *L. reuteri* reduced the detrimental effect of *E. coli* enterotoxins on the mucosal barrier [40]. ETECs can disturb the TJs between epithelial cells [9, 41, 42]. Claudins are the backbone of TJs and can modulate the paracellular route of transport [43]. Nassour and Dubreuil demonstrated that the elevated intracellular Ca^2+^ levels in response to STb enterotoxins redistribute claudin-1 from the plasma membrane to the cytosol, leading to an increase in paracellular permeability [44]. Similarly, in the current study, we found upregulation of the CLDN2 and CLDN2D genes in *E. coli*-challenged cells, while the expression of these genes was unchanged in the EPS-treated or pretreated cells. We also observed the upregulation of the PARD6B gene in the nontreated as well as pretreated cells infected with ETEC (Figure 3). This gene (a member of the PAR6 family) encodes a protein involved in the cell polarization process [45]. JAMs are among other induced molecules that participate in the generation of the cell junctional system and TJ assembly [41]. Similarly, the F11R gene, encoding the receptor for JAM-1, was also upregulated in the ETEC-challenged cells, but its expression was unchanged in the other two groups (Figure 3). Based on our results, we deduced that EPS pretreatment can reduce the overexpression of the genes participating in the formation of TJs caused by ETEC infection.

The ECM is a highly dynamic structure constantly undergoing a remodeling process [46]. The most significant enzymes participating in remodulation are metalloproteinases, which may degrade ECM. The metalloproteinases are categorized into matrix metalloproteinases (MMPs) and the ADAM (a disintegrin and metalloprotease domain) family [47]. In the current study, the ADAM17, ADAM10, MMP14 and MMP9 genes were induced in the ETEC-challenged cells (Figure 4), whereas, except for MMP14, their expression in the EPS-treated and pretreated cells remained unchanged. Thus, it can be assumed that the EPS of *L. reuteri* could reduce the ECM degradation induced by *E. coli* infection. We also observed that the ELN gene, encoding elastin, was downregulated in the ETEC-infected cells, while in the EPS-treated or pretreated cells, it remained unchanged. A similar effect of EPS pretreatment was observed for the set of genes (ITGB3 and ITGA2B) encoding integrins (Figure 4). Integrins act as receptors mainly for extracellular matrix proteins [48], which mediate downstream gene transcription through a variety of signalling pathways, for example, via NF-κB. In the present study, the activation of NF-κB was noticed in *E. coli*-infected cells (Additional file 5). To our knowledge, no work has been published to date that explains the altered expression of integrins in IPEC-1 cells upon ETEC infection.

TLR signalling pathways play crucial roles in the regulation and activation of numerous pro-inflammatory molecules. LPS is a well-known activator of TLR4 [36]; however, recent studies have shown that EPS from lactic acid bacteria can also activate TLR4, TLR2 [5] and C-type lectin signalling pathways [7]. Activation of TLR4 induced downstream MyD88-dependent and TRIF-dependent (MyD88-independent) pathways, which in turn led to a cascade of activated molecules, such as NF-κB, activator protein-1 (AP-1), interferon regulatory factor 5 (IRF5) and IRF3 (type I interferons) and, finally, inflammatory cytokines [49] (Additional file 6). We have previously demonstrated that ETEC infection of IPEC-1 cells induces the expression of the ﻿NF-κB, IL1β, TNFα, and IL6 genes; however, EPS-L26 pretreatment before ETEC infection abates the overexpression of NF-κB and IL1β but not that of the TNFα or IL6 genes [6]. Such anti-inflammatory properties of EPS have also been demonstrated by others [5, 50, 51]. In the current work, we presumed that the NF-κB pathway was activated in the cells infected with *E. coli* because increased expression of IL2, IL4, IL6, IL12B and TNFα was found (Figure 8). A plausible anti-inflammatory effect of EPS was observed in this study, as the expression of the IL12B, TNF, CSF1 and CSF3 genes was abated in the cells pretreated before *E. coli* infection (Figure 8).

In conclusion, the transcriptomic analyses performed in this study showed differential cellular responses to ETEC infection with or without pretreatment with the EPS derived from *L. reuteri*. This study allowed us to obtain a global perspective on the induced genes and pathways involved in the cellular response to EPS and *E. coli* alone and in combination. The results indicate that the use of EPS may be a good strategy to improve intestinal homeostasis. EPS modulated the genes participating in the formation of TJs. EPS also attenuated the overexpression of genes known to be activated by LT or ST, which are associated with electrolyte loss. Furthermore, the attenuation of the expression of the genes encoding proteases was observed in the cells pretreated with EPS pretreated compared to those not pretreated before *E. coli* infection. The most relevant finding of this study is that EPS has a suppressive effect on the inflammatory response evoked by *E. coli* infection. Overall, the data map offers a comprehensive picture of the positive effect of EPS pretreatment and provides sound benchmarking for further studies, which should be conducted using other omics approaches to validate the positive effects of EPS.

## Supplementary information

**Additional file 1. Analysis of the genes expressed in the IPEC-1 cells challenged with ETEC, treated with EPS, or pretreated with EPS before ETEC challenge by using Reactome.** IPEC-1 cell +ETEC: analysis based on the gene names of 463 entities; IPEC-1 cells + EPS: analysis based on the gene names of 111 entities; and IPEC-1 cells + EPS + ETEC: analysis based on the gene names of 147 entities.

**Additional file 2. DEGs involved in the tight junction organization pathway.** DEGs found in the study are highlighted in the pathway “tight junction organization” retrieved from the KEGG database. Red indicates the genes expressed in the cells challenged with ETEC. Blue indicates genes expressed in the cells treated with EPS (note, no genes in this pathway were induced by this treatment). Green indicates genes expressed in the cells pretreated with EPS and challenged with ETEC.

**Additional file 3. DEGs involved in the ECM-receptor interaction pathway.** DEGs involved in the ECP-receptor interaction pathway (retrieved from the KEGG database) as highlighted in three experimental groups. Red indicates genes expressed in the cells challenged with ETEC. Blue indicates genes expressed in the cells treated with EPS. Green indicates genes expressed in the cells pretreated with EPS and challenged with ETEC.

**Additional file 4. DEGs involved in the CLR signalling pathway.** The KEGG results showing the C-type lectin receptor signalling pathway. DEGs were highlighted on the basis of three experimental groups. Red indicates genes expressed in the cells challenged with ETEC. Blue indicates genes expressed in the cells treated with EPS. Green indicates genes expressed in the cells pretreated with EPS and challenged with ETEC.

**Additional file 5. DEGs involved in the NF kappa B signalling pathway.** DEGs involved in the NF kappa B signalling pathway are highlighted on the basis of three experimental groups. Red indicates genes expressed in the cells challenged with ETEC. Blue indicates genes expressed in the cells treated with EPS. Green indicates genes expressed in the cells pretreated with EPS and challenged with ETEC.

**Additional file 6. DEGs involved in the TLR signalling pathway.** DEGs in the TLR signalling pathway found in three cell treatments are highlighted. Red indicates genes expressed in the cells challenged with ETEC. Blue indicates genes expressed in the cells treated with EPS. Green indicates the genes expressed in cells pretreated with EPS and challenged with ETEC.

**Additional file 7. DEGs involved in the cytokine–cytokine receptor interaction pathway.** DEGs from the cytokine–cytokine receptor interaction pathway found in the cells subjected to one of the three treatments are highlighted. Red indicates genes expressed in the cells challenged with ETEC. Blue indicates genes expressed in the cells treated with EPS. Green indicates genes expressed in the cells pretreated with EPS and challenged with ETEC.

**Additional file 8. Genes identified through RNA-seq in the downstream bioinformatics analysis.** Data sets from 1.1 to 1.3—Differentially expressed genes (DEGs) found in the cells subjected to one of the three treatments. Data sets from 1.4 to 1.7—Common genes identified among the treated cells. Data sets from 1.8 to 1.10—Molecular pathways identified in the treated cells.
