# Supplementary figures and images for: Comprehensive mapping of the cell response to E. coli infection in porcine intestinal epithelial cells pretreated with exopolysaccharide derived from Lactobacillus reuteri
Source: Vet Res. 2020 Mar 31;51:49. doi: 10.1186/s13567-020-00773-1 (PMC7106801; doi:10.1186/s13567-020-00773-1)

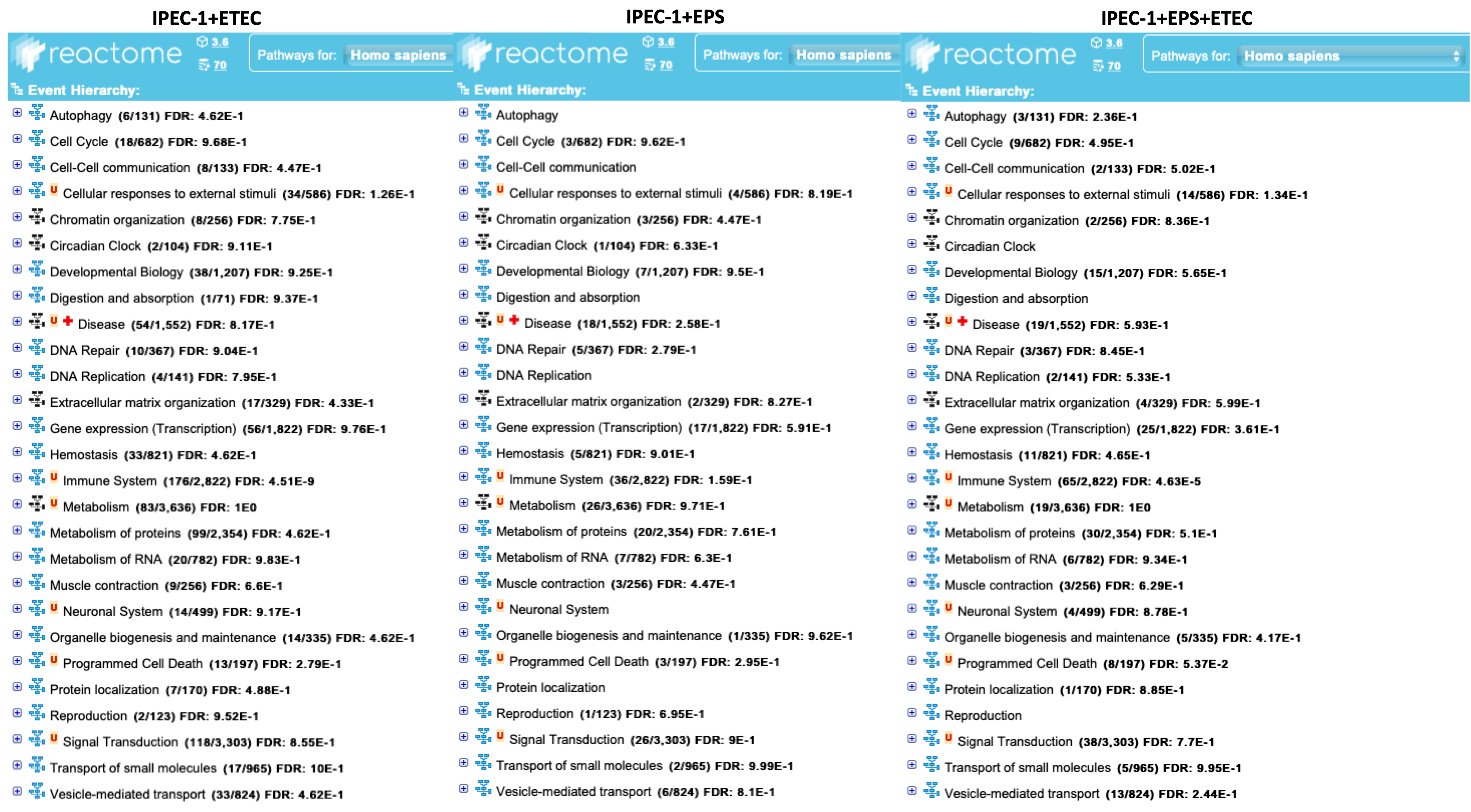

Supplement: Supplementary file 1 — Additional file 1. Analysis of the genes expressed in the IPEC-1 cells challenged with ETEC, treated with EPS, or pretreated with EPS before ETEC challenge by using Reactome. IPEC-1 cell +ETEC: analysis based on the gene names of 463 entities; IPEC-1 cells + EPS: analysis based on the gene names of 111 entities; and IPEC-1 cells + EPS + ETEC: analysis based on the gene names of 147 entities. [file 13567_2020_773_MOESM1_ESM.tiff]

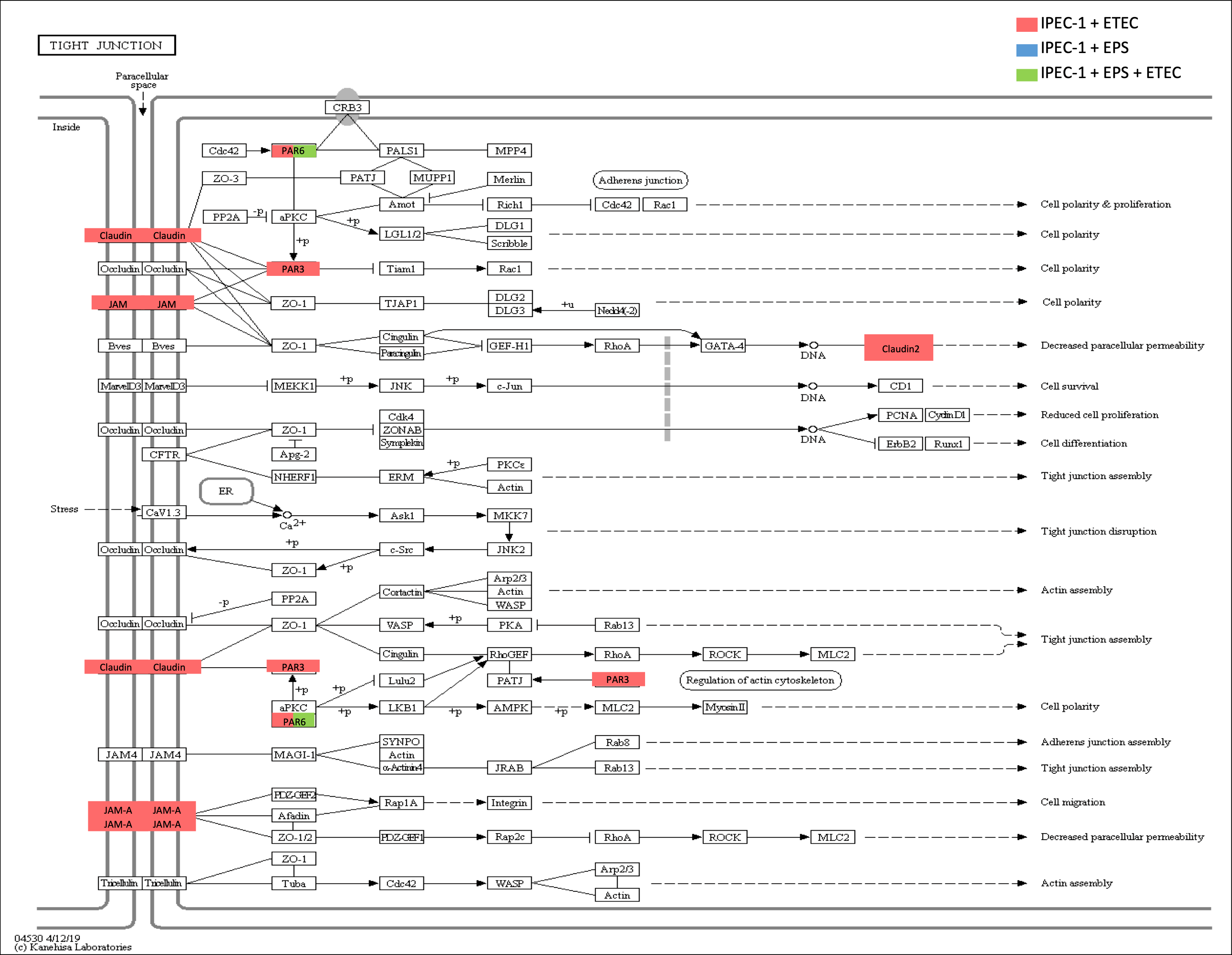

Supplement: Supplementary file 2 — Additional file 2. DEGs involved in the tight junction organization pathway. DEGs found in the study are highlighted in the pathway “tight junction organization” retrieved from the KEGG database. Red indicates the genes expressed in the cells challenged with ETEC. Blue indicates genes expressed in the cells treated with EPS (note, no genes in this pathway were induced by this treatment). Green indicates genes expressed in the cells pretreated with EPS and challenged with ETEC. [file 13567_2020_773_MOESM2_ESM.tiff]

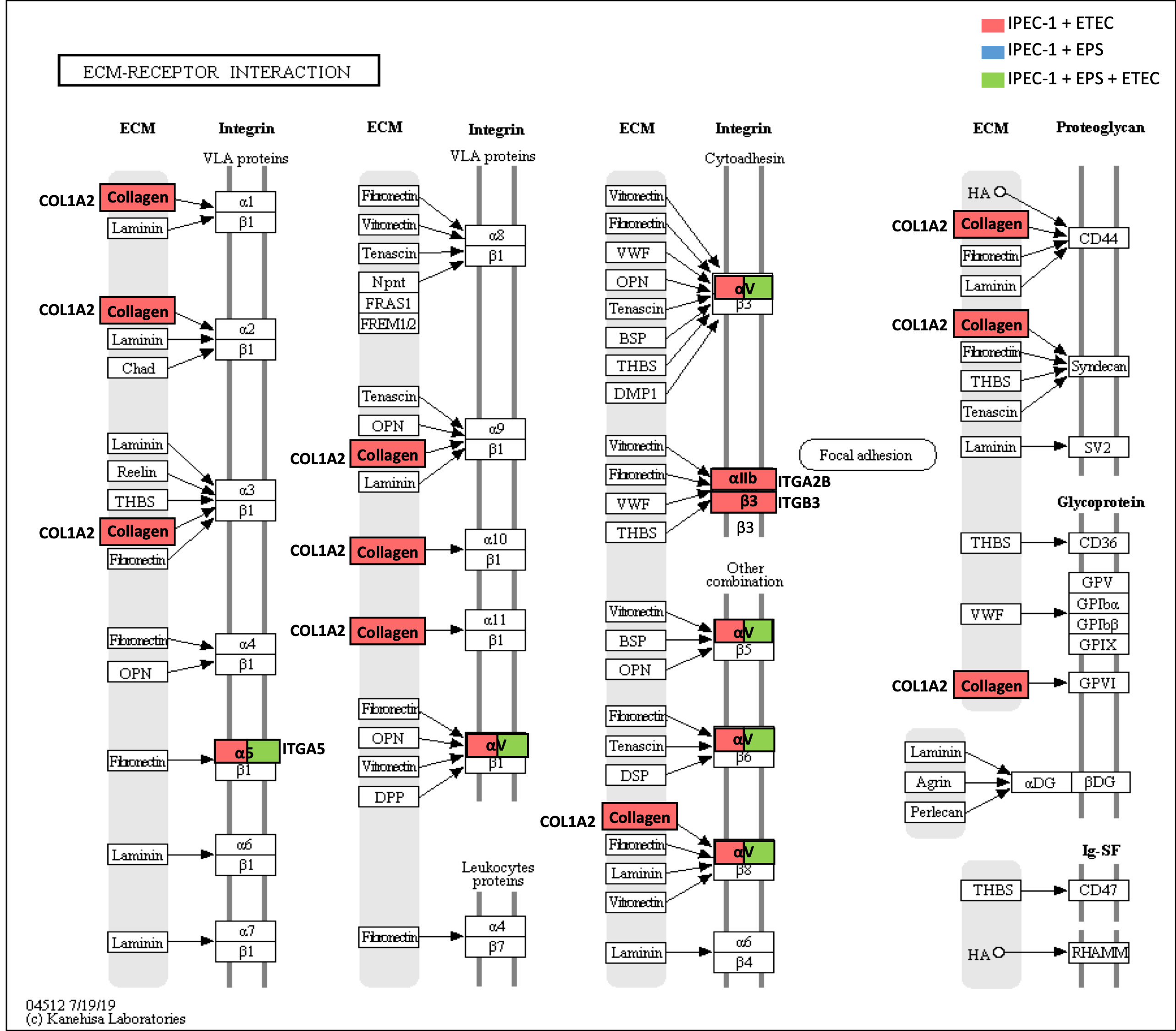

Supplement: Supplementary file 3 — Additional file 3. DEGs involved in the ECM-receptor interaction pathway. DEGs involved in the ECP-receptor interaction pathway (retrieved from the KEGG database) as highlighted in three experimental groups. Red indicates genes expressed in the cells challenged with ETEC. Blue indicates genes expressed in the cells treated with EPS. Green indicates genes expressed in the cells pretreated with EPS and challenged with ETEC. [file 13567_2020_773_MOESM3_ESM.tiff]

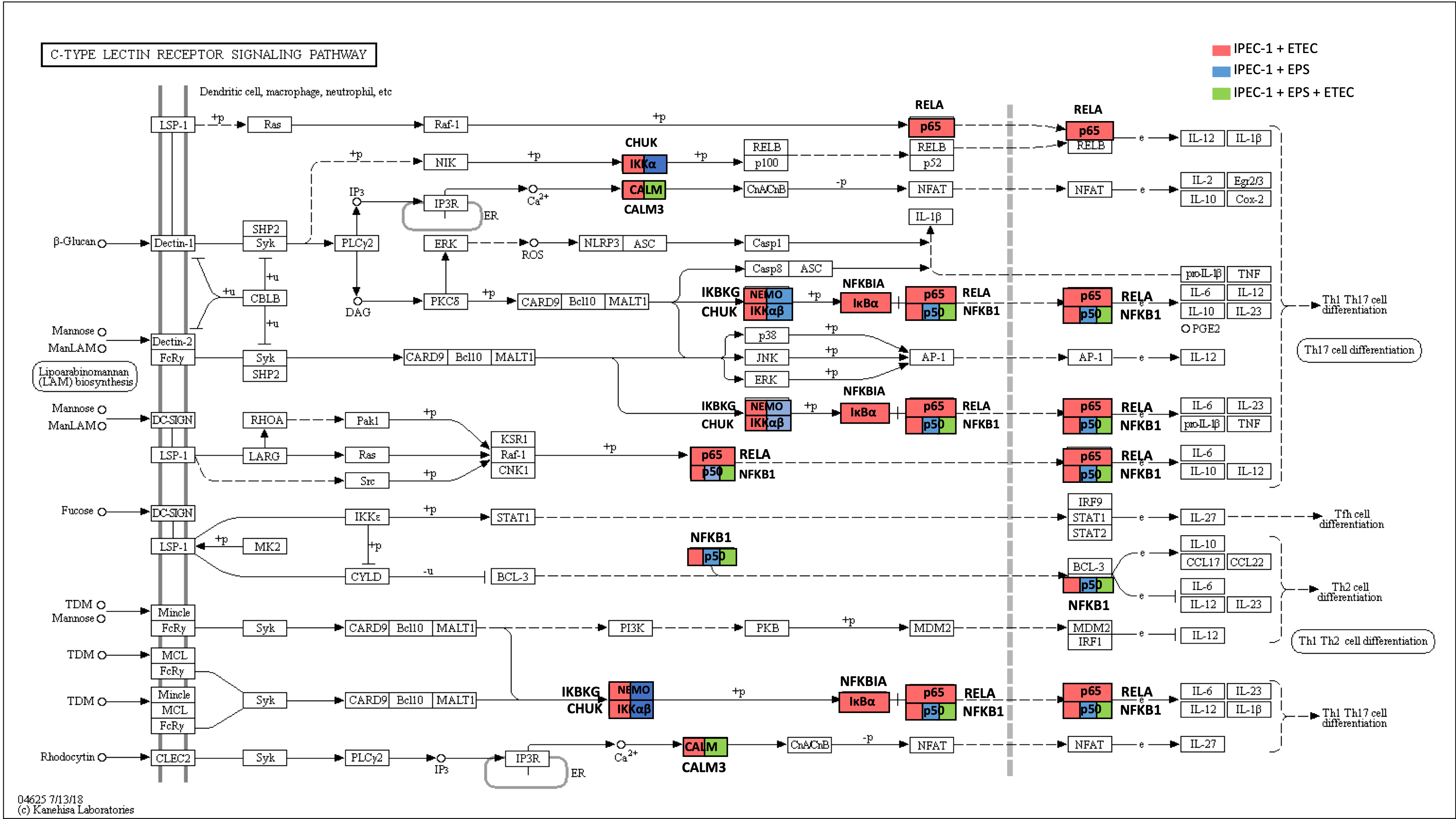

Supplement: Supplementary file 4 — Additional file 4. DEGs involved in the CLR signalling pathway. The KEGG results showing the C-type lectin receptor signalling pathway. DEGs were highlighted on the basis of three experimental groups. Red indicates genes expressed in the cells challenged with ETEC. Blue indicates genes expressed in the cells treated with EPS. Green indicates genes expressed in the cells pretreated with EPS and challenged with ETEC. [file 13567_2020_773_MOESM4_ESM.tiff]

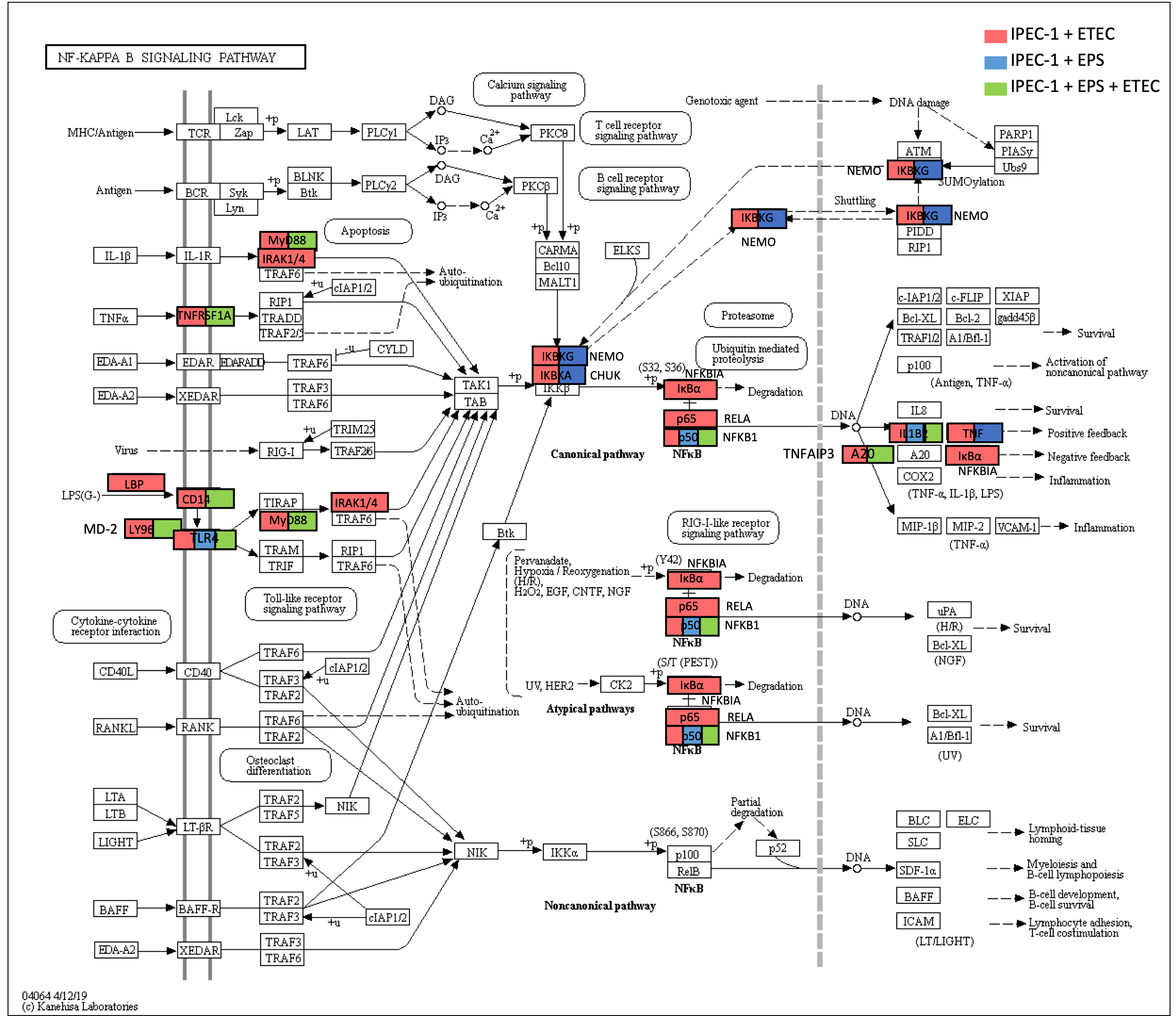

Supplement: Supplementary file 5 — Additional file 5. DEGs involved in the NF kappa B signalling pathway. DEGs involved in the NF kappa B signalling pathway are highlighted on the basis of three experimental groups. Red indicates genes expressed in the cells challenged with ETEC. Blue indicates genes expressed in the cells treated with EPS. Green indicates genes expressed in the cells pretreated with EPS and challenged with ETEC. [file 13567_2020_773_MOESM5_ESM.tiff]

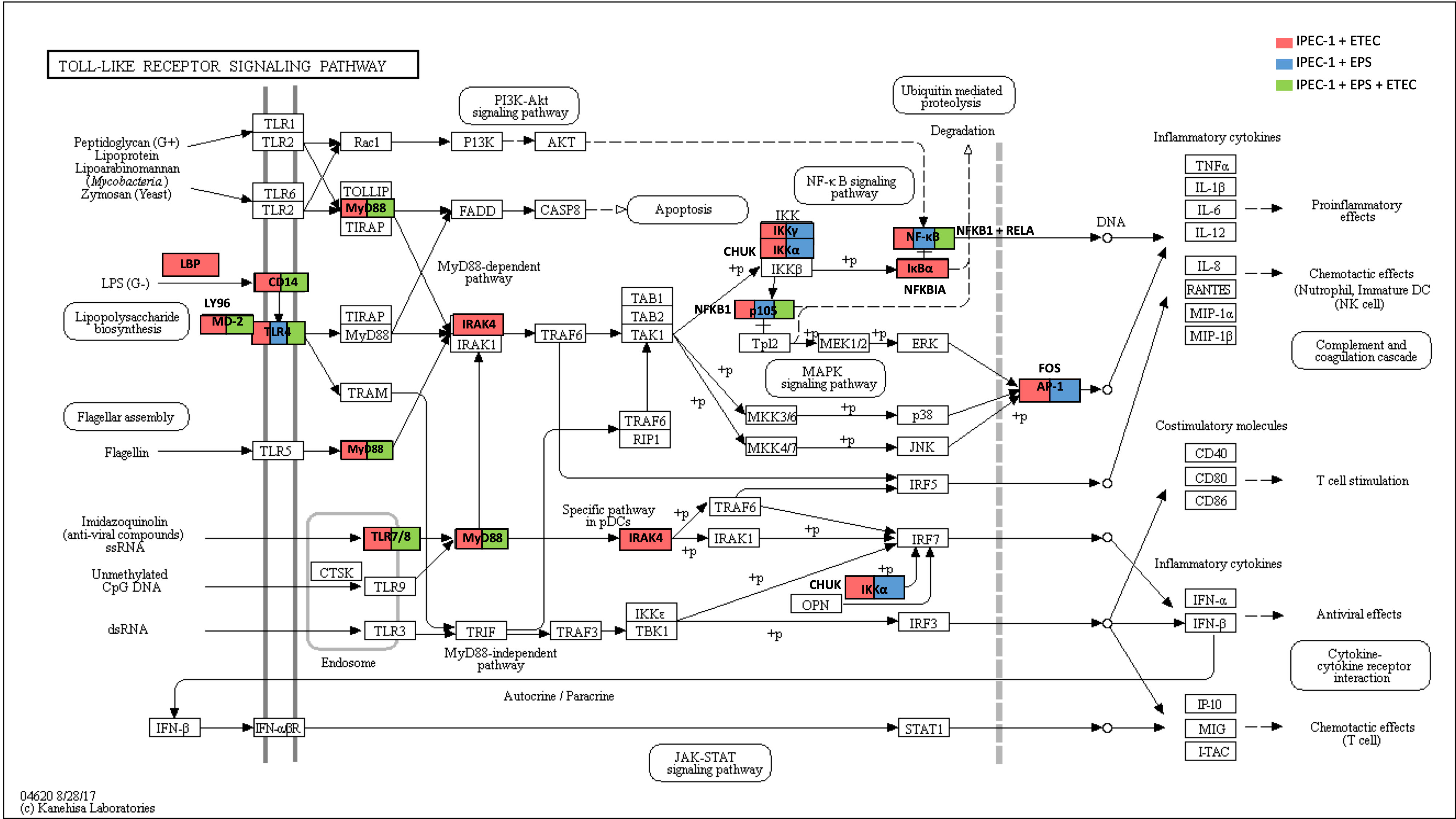

Supplement: Supplementary file 6 — Additional file 6. DEGs involved in the TLR signalling pathway. DEGs in the TLR signalling pathway found in three cell treatments are highlighted. Red indicates genes expressed in the cells challenged with ETEC. Blue indicates genes expressed in the cells treated with EPS. Green indicates the genes expressed in cells pretreated with EPS and challenged with ETEC. [file 13567_2020_773_MOESM6_ESM.tiff]

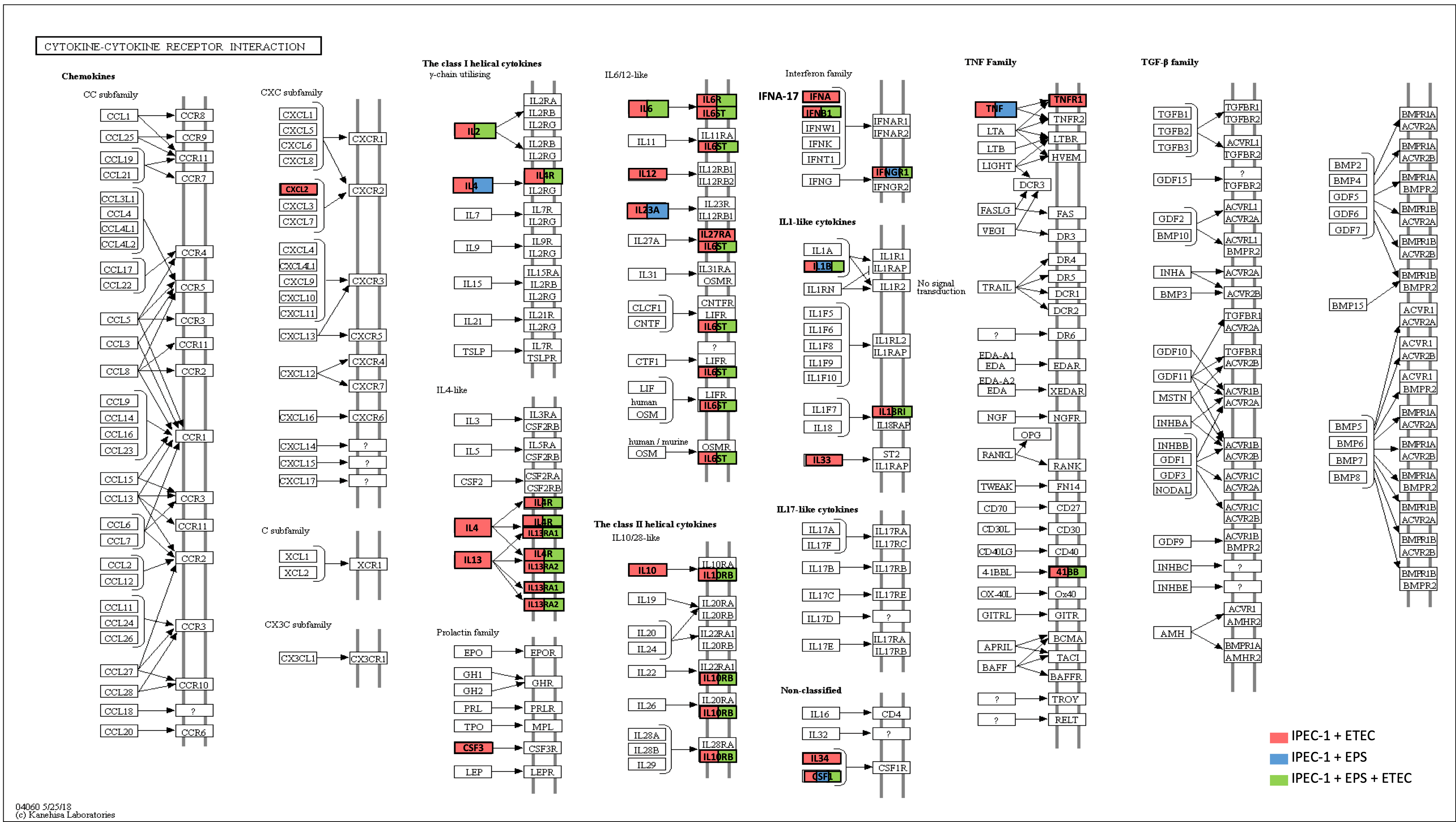

Supplement: Supplementary file 7 — Additional file 7. DEGs involved in the cytokine–cytokine receptor interaction pathway. DEGs from the cytokine–cytokine receptor interaction pathway found in the cells subjected to one of the three treatments are highlighted. Red indicates genes expressed in the cells challenged with ETEC. Blue indicates genes expressed in the cells treated with EPS. Green indicates genes expressed in the cells pretreated with EPS and challenged with ETEC. [file 13567_2020_773_MOESM7_ESM.tiff]
